# Lack of peptide YY signaling in mice disturbs gut microbiome composition in response to high‐fat diet

**DOI:** 10.1096/fj.202002215R

**Published:** 2021-03-22

**Authors:** Aitak Farzi, Chi Kin Ip, Felicia Reed, Ronaldo Enriquez, Geraldine Zenz, Marija Durdevic, Lei Zhang, Peter Holzer, Herbert Herzog

**Affiliations:** ^1^ Neuroscience Division Garvan Institute of Medical Research St. Vincent's Hospital Darlinghurst NSW Australia; ^2^ Division of Pharmacology Otto Loewi Research Center Medical University of Graz Graz Austria; ^3^ Faculty of Medicine University of NSW Sydney NSW Australia; ^4^ Center for Medical Research Medical University of Graz Graz Austria; ^5^ Diagnostic and Research Institute of Pathology Medical University of Graz Graz Austria; ^6^ Theodor Escherich Laboratory for Medical Microbiome Research Medical University of Graz Graz Austria

**Keywords:** food intake, gut hormones, intestinal barrier, intestinal microbiota, obesity

## Abstract

Peptide YY (PYY), produced by endocrine L cells in the gut, is known for its critical role in regulating gastrointestinal functions as well as satiety. However, how these processes are integrated with maintaining a healthy gut microbiome composition is unknown. Here, we show that lack of PYY in mice leads to distinct changes in gut microbiome composition that are diet‐dependent. While under chow diet only slight differences in gut microbiome composition could be observed, high‐fat diet (HFD) aggravated these differences. Specifically an increased abundance of the *Bacteroidetes* phylum with a corresponding decrease of the *Firmicutes/Bacteroidetes* ratio could be detected in *Pyy‐*knockout (KO) mice in response to HFD. Detailed analysis of the *Bacteroidetes* phylum further revealed that the *Alistipes* genus belonging to the *Rikenellaceae* family, the *Parabacteroides* belonging to the *Tannerellaceae* family, as well as *Muribaculum* were increased in *Pyy‐*KO mice. In order to investigate whether these changes are associated with changed markers of gut barrier and immunity, we analyzed the colonic expression of various pro‐inflammatory cytokines, as well as tight junction proteins and mucin 2, and identified increased mRNA expression of the tight junction proteins *Cldn2* and *Ocel1* in *Pyy‐*KO mice, while pro‐inflammatory cytokine expression was not significantly altered. Together these results highlight a critical gene‐environment interaction between diet and the gut microbiome and its impact on homeostasis of the intestinal epithelium under conditions of reduced PYY signaling which is commonly seen under obese conditions.

**Abbreviations**
Actb: actin‐β
Cldn2: claudin 2
DXA: dual‐energy X‐ray absorptiometry
FM: fat mass
HFD: high‐fat diet
LEfSe: linear discriminant analysis Effect Size
Muc2: mucin 2
NPY: neuropeptide Y
Ocel1: occludin/ELL domain containing protein 1
OTU: operational taxonomic unit
PP: pancreatic polypeptide
PCoA: principle coordinate analysis
PYY: peptide YY
SCFAs: short chain fatty acids

## 1 INTRODUCTION

The gut hormone peptide YY (PYY), a member of the neuropeptide Y (NPY) family also including pancreatic polypeptide (PP), is produced in enteroendocrine L cells of the gastrointestinal tract as well as in pancreatic islets.^1^ PYY levels increase upon ingestion of a meal proportionally to energy intake and have local paracrine, as well as endocrine effects coordinating postprandial digestion and energy homeostasis. Especially, PYY’s function as a satiety factor by signaling on Y2 receptors in the brain has attracted much attention and has been studied extensively.^2^ On the contrary, its local auto‐ and paracrine actions are less explored. Recently, the gut microbiota has emerged as a crucial regulator of PYY secretion. Specifically, gut microbiota ferment ingested dietary fiber to produce short‐chain fatty acids (SCFAs), which through activation of free fatty acid receptors expressed on enteroendocrine L cells induce PYY release.^3^, ^4^ In addition, intestinal *E coli* stationary phase proteins increase plasma PYY,^5^ further suggesting signaling pathways between the intestinal microbiota and PYY.

The paracrine actions of PYY in the intestine involve a variety of inhibitory functions that lead to the decrease of intestinal motility, anion and electrolyte secretion, thereby facilitating nutrient absorption.^1^ PYY fulfills this epithelial antisecretory effects via activation of basolateral Y1 and Y2 receptors via mechanisms including cAMP‐dependent Cl^‐^ secretion into the lumen,^6^, ^7^ suggesting that lack of PYY signaling may strongly impact on nutrient resorption as well as gut microbiome composition.

While the pathogenesis of obesity involves complex interactions of genetic and environmental factors, the increased consumption of calorie‐dense diets contributes strongly to the current obesity epidemic. With an increase in food intake and the development of obesity, not only the levels of PYY in the serum are reduced, but also the magnitude in increase upon ingestion of food is significantly lower.^8^, ^9^ The accompanied change in food preference in obesity away from diets high in fiber could be one of the contributors to this decrease in PYY levels.^10^ Going hand in hand with a change in diet, the gut microbiota is also significantly altered, probably further influencing the expression and release of PYY.^11^ However, while these findings suggest a signaling pathway between the intestinal microbiota and PYY, the effects of gut‐derived PYY on gut microbiome composition have not been investigated in detail. This work therefore aimed at exploring the impact of lack of PYY signaling on microbiome composition as well as homeostasis of the intestinal epithelium and how these processes are influenced by a dietary challenge with high‐fat diet (HFD).

## 2 MATERIALS AND METHODS

### 2.1 Animal models and maintenance

All experimental and animal care procedures were approved by the Garvan Institute/St. Vincent's Hospital Animal Ethics Committee and were conducted in agreement with the Australian Code of Practice for the Care and Use of Animals for Scientific Purposes. Male mice were used for all experiments and were housed under conditions of controlled temperature (22°C) and illumination (12:12 hr light‐dark cycle, lights on at 07:00 am). *Pyy*‐knockout (KO) mice were generated by removing the entire coding sequence including the initiation start as reported previously.^12^ Being originally generated on a C57Bl/6‐129/SvJ background, the mice were backcrossed onto the C57BL/6 strain for at least four generations. Importantly, *Pyy‐*KO mice and *Pyy*‐expressing controls were littermates co‐housed in the same cages, in order to minimize effects of maternal transmission and housing conditions on microbiome composition.^13^ Mice were provided with ad libitum access to water and a standard chow diet (8% calories from fat, 21% calories from protein, 71% calories from carbohydrate, 2.6 kcal/g; Gordon's Specialty Stock Feeds, Yanderra, NSW, Australia). At 12 weeks of age, mice were subjected to a HFD (43% calories from fat, 17% calories from protein, and 20 MJ/kg; Gordon's Specialty Feeds, Glen Forrest, WA, Australia).

Bodyweight was monitored weekly from 6 to 16 weeks of age. Basal food and water intake were determined at 11 and 16 weeks of age in *Pyy‐*KO and *Pyy‐*expressing control mice.

### 2.2 Body composition and bone densitometry analysis

All mice from both chow‐ and HFD‐fed groups were subjected to an initial body composition analysis using the dual‐energy X‐ray absorptiometry (DXA, Lunar PIXImus2 mouse densitometer; GE Healthcare, Waukesha, WI, USA) system at 8 weeks of age. A second DXA scan was performed at 15 weeks of age. Animals were anesthetized with isoflurane for the scanning procedure to determine fat mass. The head of the animal was excluded while the tail was included for the analysis.

### 2.3 Tissue collection and analysis

To collect fecal samples, mice were placed in an empty clean cage without bedding and freshly dropped fecal pellets were collected into sterile tubes using a clean tweezer.^14^ Fecal samples were stored at −80°C until analysis. Following completion of studies, all mice were sacrificed at 16 weeks of age. A 1‐cm segment of the distal colon was opened longitudinally, washed in autoclaved phosphate‐buffered saline (PBS), and shock‐frozen in dry ice for quantitative polymerase chain reaction (qPCR).

### 2.4 RNA isolation and quantitative real‐time PCR

Colonic RNA was extracted using an RNeasy Micro Kit (Qiagen) following the manufacturer's instructions. RNA quantification and purity were confirmed by NanoDrop Spectrophotometers. cDNA was synthesized from 100 ng RNA by using the SuperScript III First‐Strand Synthesis System (Thermo Fisher Scientific). RT‐qPCR using primers^15^ for various tight junction proteins and pro‐inflammatory cytokines (Table 1) was carried out from 1:5 dilution of cDNA from each sample using the LightCycler (Light‐Cycler 480 Real‐Time PCR system, Roche Applied Science, Germany), SYBR Green I (Molecular Probes), and Platinum Taq DNA Polymerase (Invitrogen). The previously described PCR condition was used in all the RT‐qPCR experiments, 94°C for 30 seconds, 62°C for 30 seconds, and 72°C for 20 seconds for 40 cycles.^16^ Expression of the gene was normalized to the expression of the housekeeping gene actin‐β (*Actb*) (Table 1).

**TABLE 1:** Primers used for assaying tight junction protein and pro‐inflammatory cytokine expression

Target genes	Forward primer	Reverse primer
*Actb*	AGCACCCTGTGCTGCTCA	GTACGACCAGAGGCATACA
*Muc2*	AAGTGTCCTTGCATCCACAA	AGATAGAGCAGGTGCTGTG
*Cldn1*	CACAGCATGGTATGGAAACA	TGGGTAAGAGGTTGTTTTCC
*Cldn2*	ATACTACCCTTTAGCCCTGACCGAGA	CAGTAGGAGCACACATAACAGCTACCAC
*Ocel1*	GAGAGTTTGAGAAGAAGCGA	AGAGTCTTCACTGTTGCTGT
*Ocln*	AGACTACACGACAGGTGGGG	CTGCAGACCTGCATCAAAAT
*Tjp1*	GCAGACTTCTGGAGGTTTCG	CTTGCCAACTTTTCTCTGGC
*Ifng*	ATCTTGGCTTTGCAGCTCTT	AGTTCCTCCAGATATCCAAG
*Il6*	CACTTCACAAGTCGGAGGCT	CTGCAAGTGCATCATCGTTGT
*Il1b*	CCCAAAAGATGAAGGGCTGC	AAGGTCCACGGGAAAGACAC
*Il18*	CCTCTCTGTGAAGGATAGTA	ACTCCATCTTGTTGTGTCCT
*Tnf*	TGCCTATGTCTCAGCCTCTT	ATAGAACTGATGAGAGGGAG
*Nos2*	GCTACCACATTGAAGAAGCT	TAGGAAAAGACTGCACCGAA
*Npy1R*	GACTCTCACAGGCTGTCTT	TTGGTCTCACTGGACCTGT
*Npy2R*	TTTTCGGAGGCTACCAATGT	AATACAATGGGAGGTCTGCA

### 2.5 Microbiome analysis—DNA isolation and PCR amplification

DNA isolation, PCR amplification and sequencing was performed by the Australian Genome Research Facility. Intestinal contents were homogenized on a PowerLyzer Homogenizer and DNA was extracted using the DNeasy PowerSoil Kit (Mo Bio Laboratories, Inc, Carlsbad, CA, USA) according to the manufacturer's instructions. PCR amplicons were generated using the primers and conditions outlined in Table 2, using AmpliTaq Gold 360 Master Mix (Life Technologies, Australia) for the primary PCR. A secondary PCR to index the amplicons was performed with TaKaRa Taq DNA Polymerase (Clontech). The resulting amplicons were measured by fluorometry (Invitrogen PicoGreen) and normalized. The equimolar pool was then measured by qPCR (KAPA) followed by sequencing on the Illumina MiSeq (San Diego, CA, USA) with 2 x 300 base pairs paired‐end chemistry. The data set was processed using the QIIME2 tool on the local Galaxy instance (https://galaxy.medunigraz.at/). Dada2 denoise‐paired algorithm was used for quality filtering, sequencing error corrections, joining reads, chimeras removing, and dereplication. Taxonomic assignment was done based on the Silva 132 database with Naïve Bayes classifier. Diversity analyses were performed on a nonsummarized Amplicon Sequence Variance (ASV) level. Samples were rarefied to 11.989 sequences per sample for Alpha and Beta Diversity calculations.^17^ The web‐based software Calypso (http://cgenome.net/wiki/index.php/Calypso) was used for normalization (total sum normalization), transformation (square root transformation), and analysis of the taxonomic data.^18^

**TABLE 2:** Primers and conditions used for PCR amplification of hypervariable region V1‐V3

Target	Cycle	Initial	Disassociate	Anneal	Extension	Finish
16S: V1‐ V3	29	95°C for 7 min	94°C for 45 s	50°C for 60 s	72°C for 60 s	72°C for 7 min
Target	27F – 519R					
Forward Primer (27F)	AGAGTTTGATCMTGGCTCAG					
Reverse Primer (519R)	GWATTACCGCGGCKGCTG					

### 2.6 Statistical analysis

Data are presented as means ± SEM. Differences among mouse groups of various genotypes and treatments were assessed by Student's t test, ANOVA or repeated‐measures ANOVA combined with Sidak post hoc analysis where appropriate. Pearson's correlation was calculated for bacterial taxa at genus level that differed between *Pyy*‐KO and control mice versus gut barrier markers followed by Bonferroni correction. Statistical analyses were performed with GraphPad Prism 6 (GraphPad Software, Inc CA, USA). Linear discriminant analysis (LDA) Effect Size (LEfSe) calculations implemented in Galaxy were used to identify the bacterial communities that differentiate diet and genotype effects.^19^ Statistical significance was defined as *P* ≤.05.

## 3 RESULTS

### 3.1 Effects of high‐fat diet on gut microbiome composition in WT and *Pyy*‐KO mice

In order to assess the impact of lack of PYY signaling on microbiome composition, we used our germline *Pyy*‐KO model^12^ and fed male mice and their littermate controls expressing *Pyy* a standard chow diet from weaning until the age of 12 weeks when they were changed over to a HFD. To assess the effects of *Pyy*‐deletion on gut microbiome composition, we analyzed the sequences of bacterial 16S rRNA gene amplicons before and after HFD feeding.

**FIGURE 1:**
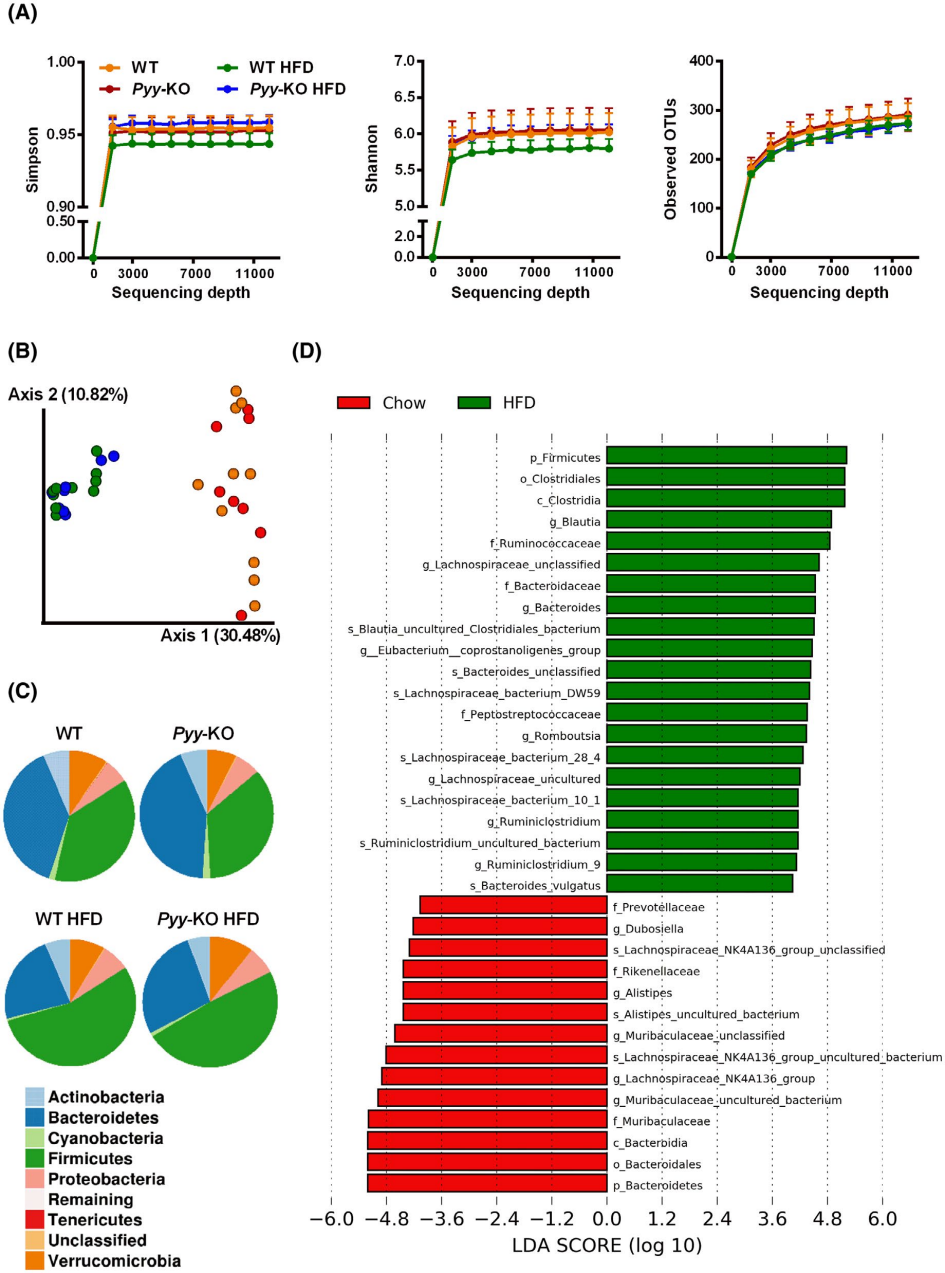
High‐fat diet has a bigger influence on microbiome composition as compared to PYY‐deficiency. Bacterial alpha diversity as assessed by Simpson, Shannon and operational taxonomic units (OTUs) (A). Bacterial communities of controls and *Pyy‐*KO mice under chow‐fed conditions as well as after 4‐week HFD feeding were clustered using Bray Curtis distance‐based principal coordinates analysis (PCoA). The percentage variation in the plotted principal coordinate (PC) is indicated on the axes (B). Pie charts of the average relative abundances of the bacterial phyla in controls and *Pyy‐*KO mice on standard chow as well as HFD (C). Most differentially abundant taxa selected by Linear discriminant analysis Effect Size (LEfSe) for diet (D). Green taxa with positive LDA scores enriched in response to HFD. Red taxa with negative LDA scores enriched in response to standard chow diet (D). n = 8‐10

Neither *Pyy‐*KO nor diet induced changes in alpha diversity, as assessed by Simpson, Shannon and operational taxonomic units (OTUs) (Figure 1A). Using principle coordinate analysis (PCoA) based on Bray Curtis, we compared fecal microbiome composition of controls and *Pyy‐*KO mice in response to control diet and HFD and could observe significant shifts in the composition in response to HFD, while no significant microbial community changes were induced as a result of PYY‐deficiency (Figure 1B). The average relative abundances of bacterial phyla illustrate increases of the phylum *Firmicutes* and decreases of the phylum *Bacteroidetes* in both WT and *Pyy‐*KO mice in response to HFD (Figure 1C). We further used LEfSe analysis to determine the key variables that separated the gut microbiome in response to HFD irrespective of genotype and identified key differentially abundant taxa between standard chow diet (negative LDA score) and HFD‐fed (positive LDA score) groups (Figure 1D).

### 3.2 Effects of PYY‐deficiency on gut microbiome composition in response to high‐fat diet

**FIGURE 2:**
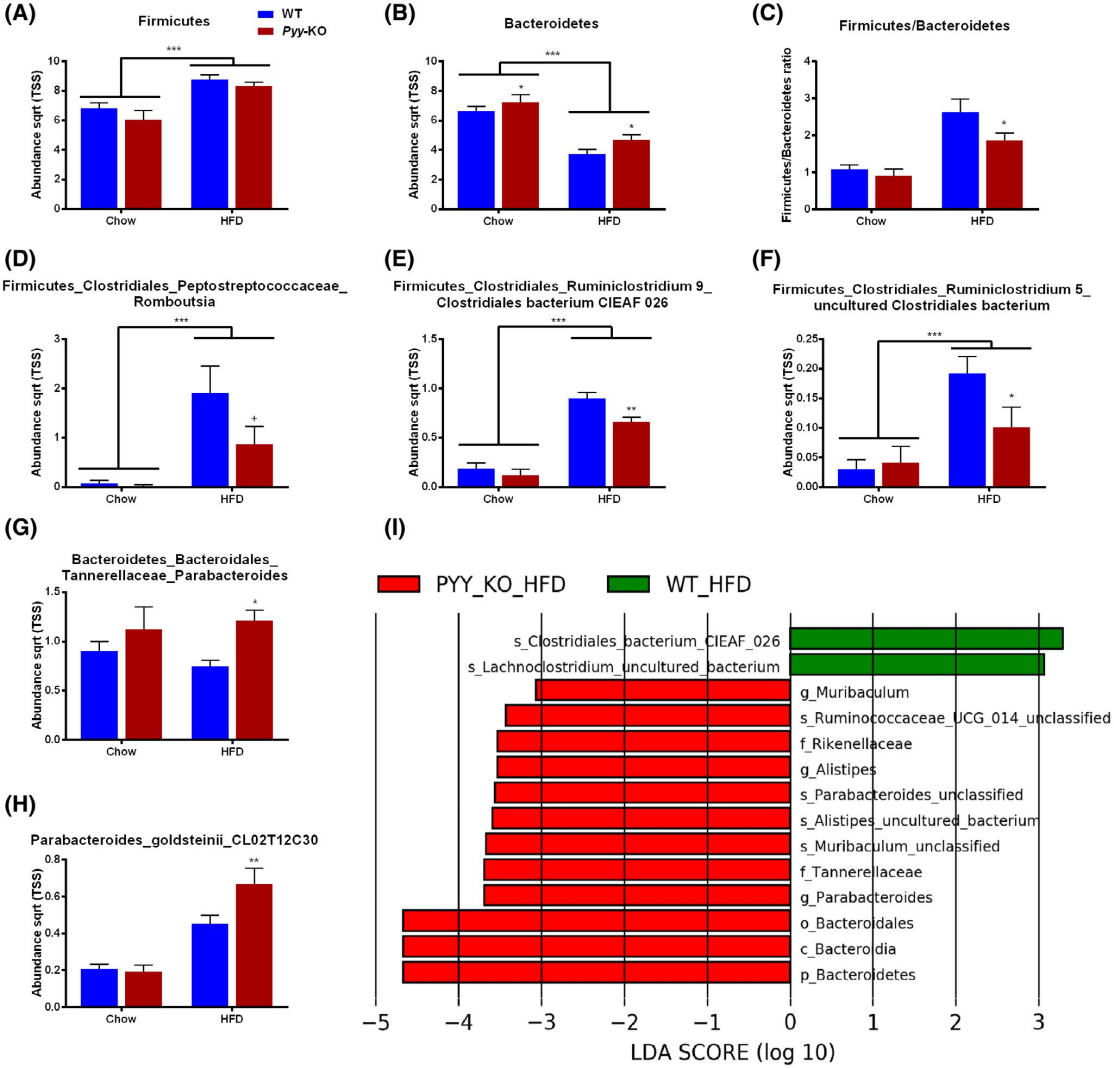
PYY‐deficiency has an impact on fecal microbiome composition in response to high‐fat diet. Abundance (total sum normalization combined with square root transformation) of the bacterial phyla *Firmicutes* (A) and *Bacteroidetes* (B), and *Firmicutes/Bacteroidetes* ratio (C). Bacterial taxa belonging to *Firmicutes* that are differentially affected by *Pyy‐*KO in response to HFD (D, E, F). Bacterial taxa belonging to *Bacteroidetes* that are differentially affected by *Pyy‐*KO in response to HFD (G, H). Most differentially abundant taxa selected by Linear discriminant analysis Effect Size (LEfSe) for genotype in response to HFD (I). Green taxa with positive LDA scores enriched in control mice in response to HFD. Red taxa with negative LDA scores enriched in *Pyy‐*KO mice in response to HFD (I). Data are means ± SEM. ^+^ *P* ≤.1; *P ≤.05; **P ≤.01; ***P ≤.001 for *Pyy‐*KO versus controls on the same diet or as indicated. n = 8‐10

In order to assess the impact of PYY signaling on microbiome composition, we screened for differentially abundant taxa in *Pyy‐*KO mice and their littermate controls expressing PYY. While there was no difference between control and *Pyy‐*KO mice for *Firmicutes* abundance (Figure 2A), *Bacteroidetes* abundance was increased in *Pyy‐*KO mice, irrespective of the diet (Figure 2B). These dynamics led to a decreased *Firmicutes/Bacteroidetes* ratio in *Pyy‐*KO mice in response to HFD as compared to control mice (Figure 2C). Analysis of the abundance of bacterial taxa at lower taxonomic ranks revealed that several taxa belonging to *Firmicutes* were lower in *Pyy‐*KO mice as compared to their littermate controls in response to HFD (Figure 2D‐F), while taxa belonging to *Bacteroidetes* were increased in *Pyy‐*KO mice (Figure 2G,H). The LEfSe method was applied to identify the key phylotypes affected by PYY‐deficiency. While the fecal microbiome of *Pyy‐*KO mice on standard chow diet was solely characterized by *Enterococcaceae* (data not shown), HFD feeding led to distinct changes in microbiome composition in *Pyy‐*KO mice (negative LDA scores) as compared to their *Pyy*‐expressing littermates (positive LDA scores). Thus, fecal microbiome of *Pyy‐*KO mice on HFD was characterized by various taxa of the *Bacteroidetes* phylum including *Alistipes* belonging to the *Rikenellaceae* family, *Parabacteroides* belonging to the *Tannerellaceae* family, as well as *Muribaculum*, while *Clostridiales bacterium CIEAF 026* and *Lachnoclostridium,* both belonging to *Firmicutes* phylum, were enriched in control mice (Figure 2I).

### 3.3 Effects of high‐fat diet on food intake, bodyweight, and fat mass in *Pyy‐*KO mice

**FIGURE 3:**
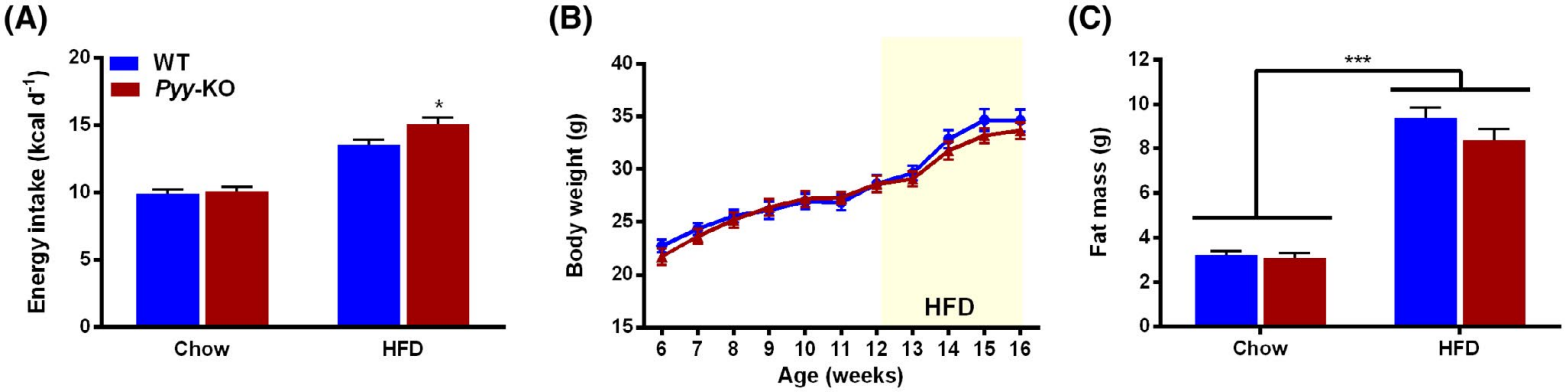
*Pyy*‐knockout increases food intake without affecting fat mass in response to high‐fat diet. Daily spontaneous/basal food intake during fed state expressed as kcal d^‐1^ in control and *Pyy‐*KO mice under standard chow and HFD (A). Absolute bodyweight measured weekly in controls and *Pyy‐*KO mice (B). Whole body fat mass determined by DXA under standard chow and HFD (C). Data are means ± SEM. *P ≤.05; ***P ≤.001 for *Pyy‐*KO versus controls on the same diet or as indicated. n = 8‐10

Bodyweight and body composition as well as food intake were monitored over the entire period in order to screen for metabolic effects of PYY‐deficiency. In line with PYY’s satiogenic effect *Pyy‐*KO mice displayed increased food intake in response to HFD (Figure 3A), while the increase in overall body weight and fat mass in response to HFD were comparable between controls and *Pyy‐*KO mice (Figure 3B,C).

### 3.4 *Pyy‐*KO is associated with higher expression of tight junction proteins

**FIGURE 4:**
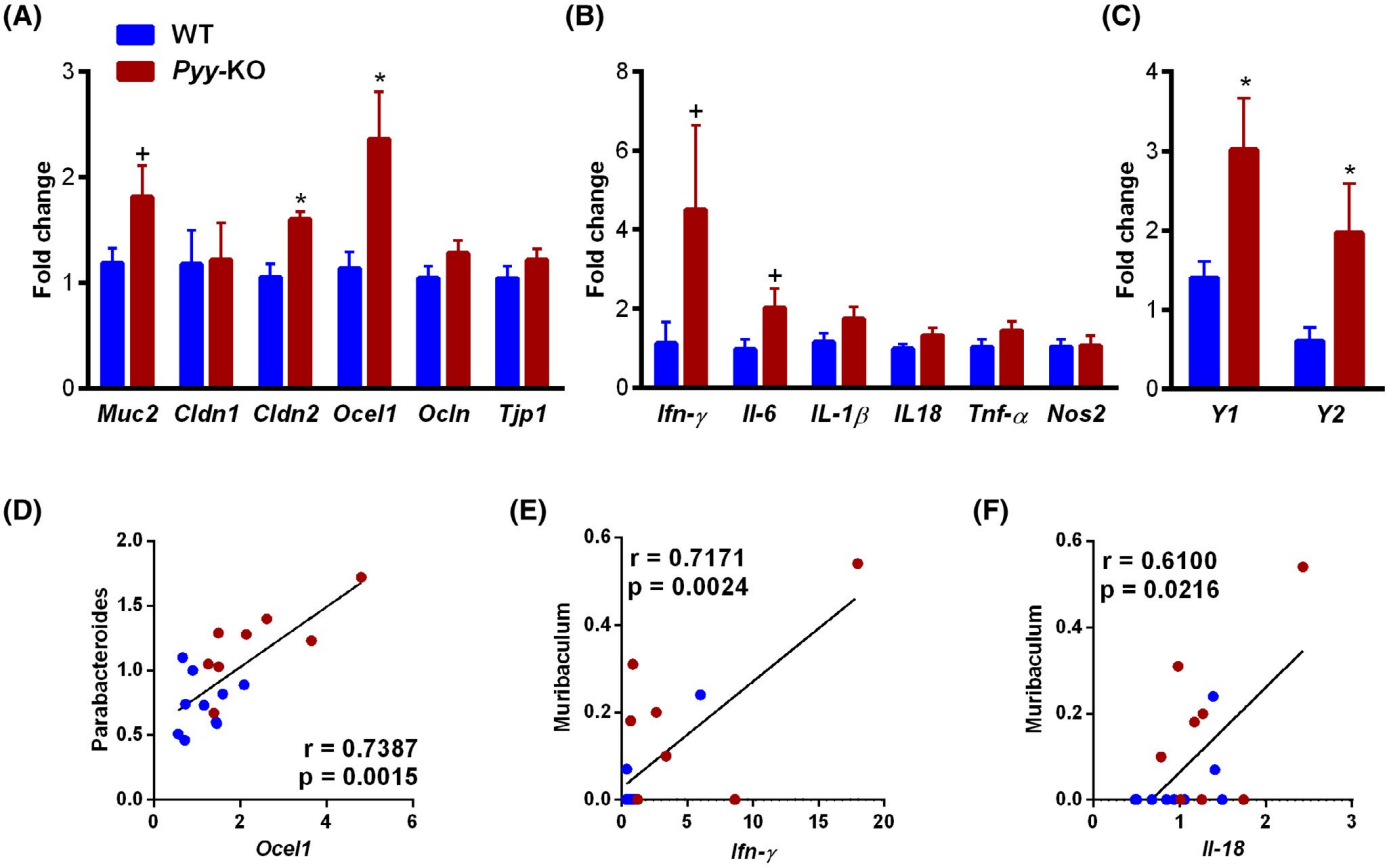
PYY‐deficiency increases *Cldn2, Ocel1,* and *Y‐receptor* mRNA expression. mRNA expression of *Muc2* and tight junction proteins (A), pro‐inflammatory cytokines (B), and Y‐receptors (C). Correlations of abundance of bacterial taxa with *Ocel1* (D), *Ifn‐ɣ* (E), and Il‐18 (F). Data are means ± SEM. ^+^ *P* ≤.1; *P ≤.05 for *Pyy‐*KO versus controls. n = 8‐10

Given the changes in gut microbiome composition in *Pyy‐*KO mice in response to HFD, we next assessed whether these changes are associated with changes in the expression of mucin 2 (*Muc2*), tight junction proteins, and pro‐inflammatory cytokines. Interestingly, we could observe an increase in mRNA expression of the tight junction proteins Occludin/ELL Domain Containing Protein 1 (*Ocel1)* and Claudin 2 (*Cldn2*) in *Pyy‐*KO mice, while the expression of other tight junction proteins was left unchanged (Figure 4A). We further observed a trend toward increased expression of the pro‐inflammatory cytokines interferon‐ɣ (*Ifn‐ɣ*; *P* =.1) and interleukin‐6 (*Il‐6*; *P* =.057) (Figure 4B).

In order to assess how lack of PYY affects the expression of its main intestinal receptors, we analyzed the expression of *Y1* and *Y2* receptors (Figure 4C). As expected, the expression of these receptors increased in the absence of their ligand PYY.

Correlation analyses were further performed based on the bacterial taxa that differed at genus level. A positive correlation between *Parabacteroides* and *Ocel1* was evident (Figure 4D), while *Muribaculum* displayed positive correlations with *Ifn‐ɣ* and *Il‐18* (Figure 4E,F).

## 4 DISCUSSION

This study demonstrates that in addition to the well‐known intestinal effects of PYY as an antisecretory agent and being a mediator of the ileal brake, PYY also controls microbiome composition since lack of PYY leads to strong dysregulation. This is especially obvious in response to a dietary challenge with HFD, which is also associated with alterations in the expression of markers of intestinal barrier function. While diet had a bigger impact on microbiome composition as compared to genotype, confirming previous findings,^20^ we could demonstrate that a 4‐week HFD feeding also leads to distinct changes in gut microbiome composition in *Pyy‐*KO mice, as compared to their littermate controls expressing PYY. Fecal microbiome composition of HFD‐fed *Pyy‐*KO mice was characterized by increased abundance of bacterial taxa belonging to the *Bacteroidetes* phylum (*Tannerellaceae_Parabacteroides, Rikenellaceae_Alistipes, Muribaculum*), while taxa of the *Firmicutes* phylum (*Ruminiclostridium_Clostridiales bacterium, Lachnoclostridium_uncultured bacterium*) were enriched in WT mice in response to HFD. Accordingly, the characteristic increase of the *Firmicutes/Bacteroidetes* ratio in response to HFD was blunted in *Pyy‐*KO mice. The observed changes in microbiome composition were associated with increased expression levels of the pore‐forming tight junction protein *Cldn2*, as well as *Ocel1* in *Pyy‐*KO mice.^21^

In line with PYY’s ability to induce the ileal brake and inhibit epithelial electrolyte secretion, upper intestinal transit has been demonstrated to be faster in *Pyy‐*KO mice, being one plausible reason for the observed changes in gut microbiome composition in *Pyy‐*KO mice.^22^ Furthermore, a polyethylene glycol‐induced increase in gut motility has been shown to lead to increased abundance of *Bacteroidaceae*,^23^, ^24^ while a morphine‐induced decrease of gut motility and secretion leads to a decreased abundance of *Bacteroidales*.^25^

More detailed analysis of the *Bacteroidetes* phylum revealed that the *Alistipes* genus belonging to the *Rikenellaceae* family, *Parabacteroides* belonging to the *Tannerellaceae* family, as well as *Muribaculum* were increased in *Pyy‐*KO mice, especially in response to HFD. *Alistipes* represents a relatively new bacterial genus being implicated in the modulation of colitis and colorectal cancer, among others.^26^ Interestingly, *Alistipes* and *Muribaculum* have a similar pattern of functional distribution as demonstrated by metagenomic and metatranscriptomic analyses.^27^ Taxa belonging to the *Muribaculum* genus, previously classified as *S24‐7*, are specialized in bile acid deconjugation, among others^28^, ^29^ and have been reported to be decreased in constipation.^30^ In the present study, we observed a positive correlation between the genus *Muribaculum* and colonic pro‐inflammatory cytokine expression. In line with this finding*, Muribaculum* has been reported to be overrepresented in mice developing inflammation in the T‐cell transfer model of chronic colitis,^31^ suggesting a potential pro‐inflammatory capacity of this genus. In general, despite its high genomic diversity, the *Bacteroidetes* phylum is characterized by a high polysaccharide utilization capacity, which enables them to degrade both dietary and host mucosal glycans.^32^, ^33^ Interestingly, a fiber‐free diet has been demonstrated to induce an increase of mucus‐degrading bacteria, leading to mucus degradation and a compensatory increase in Muc2 expression,^34^ which is critical for the formation of the intestinal mucus barrier.^35^ Importantly, an upregulation of Muc2 expression in colon epithelial cells has also been reported to be induced by pathogens as well as pro‐inflammatory cytokines.^36^, ^37^

In addition to the mucus barrier, tight junction proteins are important for maintaining a selective barrier function. While several tight junction proteins decrease epithelial permeability, Cldn2 is a pore‐forming tight junction protein creating cation selective pores leading to increased permeability of the intestinal epithelium.^38^ Cldn2 expression is upregulated by pro‐inflammatory cytokines and shows higher expression under inflammatory conditions such as inflammatory bowel disease.^39^ Similarly, Ocel1 has been demonstrated to be necessary for cytokine‐induced occludin mobilization, internalization, and barrier loss^21^ and has been proposed to enhance epithelial apoptosis through increased caspase‐3 transcription.^40^ In an attempt to deduce potential associations between differentially changed microbial taxa and altered colonic barrier markers, we screened for significant correlations and observed positive correlations between *Parabacteroides* and *Ocel1*. In line with this finding, increased abundance of *Parabacteroides* has been associated with a penetrable mucus barrier.^41^, ^42^, ^43^

As expected, expression of intestinal Y‐receptors was increased due to knockout of their ligand PYY.^44^ Interestingly, a recent publication investigated interactions between metabolites of the intestinal microbiota and various receptors including Y1 and Y2.^45^ No agonism could be demonstrated between microbial metabolites and Y1 or Y2 receptors. We therefore hypothesize that upregulation of Y1 and Y2 receptors per se does not affect interactions between microbial metabolites and Y‐receptors.

Postprandial release of PYY has been repeatedly reported to be attenuated in obese patients.^46^ On the contrary, caloric restriction is able to ameliorate the blunted PYY‐release seen in obesity leading to increased PYY‐release in response to a high‐fat meal,^47^ while increased postprandial secretion of PYY after Roux‐en‐Y gastric bypass contributes to appetite reduction and weight loss after surgery.^48^ Moreover, it has been demonstrated that both mice and humans show differential patterns of PYY‐release in response to a standardized meal, which correlates with satiety scores.^8^ While the underlying mechanisms of these differing responses have not been fully identified, diet‐microbiome interactions are likely to be involved.^49^, ^50^ *Pyy‐*KO mice are thus a suitable model of blunted PYY‐release as observed in obese patients.^46^ While we observed an expected increase in food intake in *Pyy‐*KO mice, bodyweight and composition were comparable in the littermates expressing PYY. This metabolic phenotype is in line with the previously published findings in *Pyy‐*KO mice, were increases in fat mass in *Pyy‐*KO as compared to WT controls usually develop after a longer period of HFD feeding as well as in response to an earlier onset of HFD feeding.^51^

In conclusion, this paper is the first to describe gut microbiome perturbations in mice lacking PYY signaling, especially demonstrating that in response to HFD this leads to increased abundance of bacterial taxa belonging to the *Bacteroidetes* phylum (*Tannerellaceae_Parabacteroides, Rikenellaceae_Alistipes,* and *Muribaculum*) and associated changes in the mRNA expression of tight junction proteins *Cldn2* and *Ocel1*. Together these results highlight a critical gene‐environment interaction between diet and the gut microbiome with the potential to affect homeostasis of the intestinal epithelium under reduced PYY signaling. Intestinal PYY signaling thus regulates gut microbiome composition and tight junction expression in response to HFD.

## CONFLICT OF INTEREST

No potential conflicts of interest associated with this article were reported and there has been no significant financial support for this work that could have influenced its outcome.

## AUTHOR CONTRIBUTIONS

A. Farzi, P. Holzer, and H. Herzog designed the research; A. Farzi, C. K. Ip, F. Reed, R. Enriquez, and G. Zenz performed research; M. Durdevic and L. Zhang analyzed data; A. Farzi and H. Herzog wrote the paper.
